# Evaluation of B cell related markers and autoantibodies in rheumatoid arthritis patients treated with abatacept

**DOI:** 10.3389/fimmu.2025.1504454

**Published:** 2025-01-24

**Authors:** Ting Wang, Natalia V. Giltiay, Christian Lood, Ning Wang, Bobby Kwanghoon Han

**Affiliations:** Division of Rheumatology, University of Washington, Seattle, WA, United States

**Keywords:** abatacept, predictive biomarker, B cell, CXCL13, rheumatoid factor, rheumatoid arthritis

## Abstract

**Objectives:**

To investigate whether biomarkers related to B cell activation and autoantibody production are associated with the response to abatacept in rheumatoid arthritis (RA) patients.

**Methods:**

Twenty-five patients with RA were enrolled in this study. Responders (n=10) to abatacept were subjects who achieved ACR50 response at week 24. Serum levels of soluble biomarkers were measured with ProcartaPlex by Luminex or ELISA. Peripheral blood mononuclear cells were isolated and analysed for T cell and B cell subsets by flow cytometry. Patients were genotyped for human leukocyte antigen (HLA)-DRB1 shared epitope (SE) alleles. Baseline levels and longitudinal changes of markers were assessed between responders and nonresponders.

**Results:**

Baseline levels of anti-cyclic citrullinated peptide (anti-CCP) antibodies (p=0.01), IgM rheumatoid factor (RF) (p=0.02), CXC chemokine ligand 13 (CXCL13, p=0.02), sCD23 (p<0.05), as well as frequencies of CD19^+^CD11c^+^IgD^-^CD27^-^ B cells (p=0.04), were higher in responders than nonresponders. Among them, anti-CCP and frequencies of CD19^+^CD11c^+^IgD^-^CD27^-^ B cells were independently associated with response to abatacept. The presence of two alleles of SE was associated with responders (p=0.04). Patients with 2 alleles of SE had higher levels of anti-CCP (p=0.02) and IgM RF (p=0.04) compared to patients with 0 or 1 allele. Further, IgM RF and CXCL13 levels decreased only in responders (p=0.02 and 0.004 respectively, at week 24), while anti-CCP levels did not decrease significantly in either responders or nonresponders.

**Conclusion:**

Markers of B cell activation including anti-CCP and frequencies of CD19^+^CD11c^+^IgD^-^CD27^-^ B cells in RA were associated with response to abatacept. IgM RF and CXCL13 decreased only in responders and could be potentially used as pharmacodynamic markers.

## Introduction

Rheumatoid arthritis (RA) is an autoimmune and inflammatory disease characterized by the involvement of multiple cells and cytokines in its pathogenesis. In the RA synovium, various cellular phenotypes have been identified, including myeloid, lymphoid, low inflammatory, and fibroid, each with a distinct gene expression signature (1). Notably, higher levels of T cells and B cells have been observed in the synovium of anti-CCP positive RA patients compared to anti-CCP negative patients (2). Cutting-edge laboratory technologies such as single-cell transcriptomics and mass cytometry analysis have further revealed several synovial phenotypes in RA (3).

Multiple advanced therapies targeting a specific pathway in the pathogenesis of RA have been developed. However, there are no reliable markers to guide the optimal use of these medications. Consequently, many patients with moderate to severe RA undergo “trial and error” process to identify the most suitable treatment option for their condition. Therefore, there is an unmet need for effective biomarkers that help assess the efficacy and predict the response of RA medications.

CXCR5^-^ PD-1^hi^ peripheral helper cells (Tph) have been described in synovium of seropositive RA patients (4), in contrast to CXCR5^+^ T follicular helper cells (Tfh), which function predominately in the lymph nodes. One of the primary functions of both Tfh and Tph cells is to promote differentiation of B cells into antibody-secreting plasma cells.

Abatacept, a fusion protein consisting of the extracellular domain of human CTLA-4 and a fragment of the Fc domain IgG1, works by blocking an interaction between CD80/CD86 on antigen presenting cells and CD28 on T cells. Abatacept has been reported to be more effective in seropositive RA patients with high baseline anti-CCP levels (5) and HLA-DRB1 risk alleles (6).

It has been shown to suppress Tfh cell maturation and proliferation in murine models of RA (7). Additionally, abatacept treatment reduced circulating Tfh cells and lowered the expression of the activation marker ICOS on T cells in patients with primary Sjogren’s syndrome (8) and multiple sclerosis (9). In RA patients, abatacept has been shown to reduce serum ACPA and RF levels and lower the frequencies of post-switch memory B cells (CD19^+^CD27^+^IgD^-^) (10). These results suggest that the inhibition of activated Tfh and Tph cells, leading to attenuated T cell-dependent B cell activation, may underlie the efficacy of abatacept. CD19+CD11c+ (IgD^-^CD27^-^) double negative 2 B cells emerged as autoreactive B cells that are activated in extrafollicular pathways (11) and are increased in patients with rheumatoid arthritis and other rheumatic diseases (12). In addition to serving a precursors of antibody secreting cells (ASCs), DN2 CD11c+ B cells have been also proposed to present antigens to T cells (13) and interact with Tfh/Tph cells (14). Still little is known about the potential effects of abatacept on suppressing DN2 B cells in connection with its direct effects on reducing Tfh and Tph cells.

Soluble markers shed from activated T cells and B cells have been reported to be increased in various inflammatory conditions including RA (15–17). Additionally, cytokines and chemokines associated with Tfh and Tph cells such as IL-21 and CXCL13 were elevated in RA (18, 19). Their levels may reflect activity of specific cell population and could be useful markers to evaluate RA inflammation. It is unknown whether these markers improve in response to abatacept.

The intent of this study is to investigate the pathways leading to B cell activation and autoantibody production and identify novel biomarkers that could be helpful in predicting and assessing the response to abatacept in RA patients who have an inadequate response to methotrexate and other conventional disease modifying anti-rheumatic drugs (cDMARDs).

## Materials and methods

### Study population

A total of 25 adult patients with RA who were recruited from the rheumatology clinics at the University of Washington, Seattle, WA, USA were enrolled between May 2019 and December 2022 (**Table 1**). All the participants fulfilled the 1987 ACR criteria or 2010 ACR/EULAR criteria for RA (20) and had clinical disease activity index (CDAI) ≥16 at screening corresponding to moderate to severe disease activity. The subjects had an inadequate response to conventional DMARDs including methotrexate, leflunomide, hydroxychloroquine, and/or sulfasalazine within the past year and were biologic-naïve except one patient who took etanercept 8 months prior to screening and stopped due to adverse events.

**Table 1:** Baseline characteristics.

	Responders	Nonresponders	p-value
Age, yrs	51.5 (17.0)	55.0 (23.0)	0.77
Female gender (%)	70	100	0.07
Disease duration, yrs	2.0 (8.0)	7.0 (10.5)	0.11
CDAI	23.8 (10.9)	32.1 (15.1)	0.10
DAS28-CRP	4.7 (0.9)	4.9 (1.6)	0.65
Anti-CCP (%)	90	62	0.18
RF (%)	90	54	0.09
ESR, mm/h	17.5 (10.0)	21.0 (28.0)	0.75
CRP, mg/L	11.1 (14.6)	4.3 (6.1)	0.07
Concomitant DMARDs use (%)	90	85	>0.99

Data displayed as median (interquartile range). Differences between responders (n=10) and nonresponders (n=13) were assessed by the Mann-Whitney test and Chi-squared test. P values < 0.05 considered significant.

All the subjects received abatacept 125mg subcutaneous injection weekly for 24 weeks and continued conventional DMARDs which they were on at screening visit. The subjects had 4 visits (baseline, week 6, week 14, and week 24) and peripheral blood samples were obtained at each visit. Responders to abatacept were defined as subjects who achieved ACR50 response at week 24. The research protocol was approved by the Institutional Review Board of the University of Washington, and all patients provided written informed consent for participation in the study.

### Flow cytometry

Peripheral blood mononuclear cells (PBMCs) were isolated with Ficoll-Hypaque (GE Healthcare Life Sciences, Marlborough, MA) as instructed. After isolation, PBMCs were stored frozen with Cell Freezing and Preservation Media (STEMCELL Technologies, Vancouver, Canada) in liquid nitrogen until further use. Vials of cryopreserved PBMC were thawed at 37°C in a water bath before immediately being diluted drop-wise into warm RPMI 1640 containing 10% FCS (Fisher Scientific, Waltham, MA). Thawed PBMCs were stained with combinations of fluorescently labeled antibodies (**Supplementary Table 1**) to identify distinct T cell and B cell subsets (**Supplementary Figures 1**, **2**). All longitudinal samples from the same subject were run on the same day. Samples from two to six subjects were run on the same day with the same setting template. Live cells were identified using the Zombie Fixable Viability Sampler Kit (BioLegend, San Diego, CA). Ten-parameter flow cytometry was performed and analysed for changes in different T cell and B cell subsets at baseline, week 14, and week 24 using a 4-laser CytoFLEX flow cytometer (Beckman Coulter, Brea, CA). An average of 300,000 live lymphocytes were collected, and samples with <50 events for evaluated populations at baseline were excluded from the longitudinal analysis. All data were analysed using FlowJo software (Tree Star, Ashland, OR).

### ELISAs

Freshly isolated serum samples were aliquoted and stored in a −80°C freezer until use. Commercial enzyme-linked immunosorbent assay (ELISA) kits were used for serum measurements of soluble CD23 according to the manufacturer’s instructions (R&D Systems, Minneapolis, MN). Anti-CCP ELISA Assay Kits (Eagle Biosciences, Amherst, NH) were used to determine the IgG levels of anti-CCP antibodies and Rheumatoid Factor IgM ELISA kits (Abcam, Cambridge, UK) were applied to measure serum IgM RF as instructed by the manufacturers. Anti-CCP and IgM RF were also measured via multiplex and turbimetry in the clinical laboratory (**Table 1**) at University of Washington Medical Center. The upper limit of anti-CCP was 300 U/mL in the clinical lab.

### Luminex immunoassays

The following biomarkers in serum, including soluble CD25, soluble CD27, soluble CD40L, soluble PD-1, CXCL10, CXCL13, and IL-21, were measured with ProcartaPlex by Luminex Immunoassays (Thermo Fisher Scientific, Waltham, MA) at the Histology and Imaging Core, University of Washington (Seattle, WA) (https://uwhistologyandimaging.org/histopathology-work-order/).

### HLA-DRB1 genotype

Human leukocyte antigen (HLA)-DRB1 shared epitope (SE) genotypes were determined using targeted next-generation sequencing on frozen PBMCs (LabCorp, Burlington, NC). Patients were considered SE-positive if they had ≥ 1 of the following HLA-DRB1 alleles (6): *01:01, *01:02, *01:05, *04:01, *04:04, *04:05, *04:08, *04:09, *04:10, *04:13, *04:16; *04:19, *04:21, *04:35, *04:66, *10:01, *14:02, *14:06, *14:09, *14:13, *14:17, *14:19, *14:20, and *14:21. Patients were classified as SE2 (2 SE allele), SE1 (1 SE allele), or SE0 (no SE alleles).

### Statistical analysis

GraphPad Prism 8.0 (GraphPad, San Diego, CA) and SPSS software were used for statistical analyses. For sample sets with a non-Gaussian distribution of values, the Mann-Whitney test and Spearman’s correlation test were used as applicable. Categorical variables were compared using Chi-squared test or Fisher’s exact test. Changes before and after treatment were compared using the Wilcoxon matched-pairs signed rank test. The associations between baseline variables and response to abatacept were estimated by Lasso regression analyses. P values less than 0.05 were considered significant.

## Results

### Clinical efficacy of abatacept

Twenty-three patients completed the study. One patient dropped out because of concerns for COVID infection and one patient dropped out for a reason that was not related to the study. The baseline characteristics of the patients are summarized in **Table 1**. Responders to abatacept were defined as subjects who achieved ACR50 response at week 24. After 24 weeks of treatment, 10 patients (43%) had ACR50 response. ACR20 response was achieved in 14 patients (61%) and ACR70 response was achieved in 3 patients (13%). Seven patients (30%) achieved DAR28-CRP remission (<2.6). There were no significant differences between responders and nonresponders though the frequency of females tended to be higher and anti-CCP and RF positivity tended to be lower among nonresponders. Consistent with prior work (5) where abatacept was more effective in seropositive RA patients with high anti-CCP levels, nine out of ten patients with ACR50 response had high baseline anti-CCP levels (>300 U/ml) and were double-positive for both anti-CCP and IgM RF (**Table 1**). In this study, 10 out of 23 subjects (43%) experienced adverse events. There were 3 upper respiratory infection, 3 headaches, 3 gastrointestinal symptoms (1 abdominal pain, 1 diarrhea, 1 intestinal infection) and 1 urinary tract infection. There were no serious adverse events and no subject discontinued abatacept because of the adverse events.

### Comparison of baseline markers between responders and nonresponders to abatacept

We compared the baseline levels of T cell and B cell related soluble and cellular markers between responders and nonresponders to assess whether these markers are associated with response to abatacept. In patients who achieved ACR 50 response at 24 weeks, baseline levels of anti-CCP, RF, CXCL13, sCD23 and frequencies of CD11c^+^IgD^-^CD27^-^ B cells were elevated as compared to patients not achieving ACR50 response (**Table 2**). The increase in sPD-1 levels among responders did not reach statistical significance (p=0.06). Baseline IL-21 levels were elevated only in patients who had ACR20 response (p=0.03, data not shown). On the other hand, there was a tendency that sCD25 were lower at baseline (p=0.09) in patients who had ACR50 response.

**Table 2:** Comparisons of baseline markers between responders and nonresponders to abatacept.

Markers	Responders	Nonresponders	p-value
Anti-CCP (U/mL)	48141 (57277)	1645 (9408.5)	0.01
RF IgM (IU/mL)	20099 (21531)	4228 (14951)	0.02
CXCL13 (pg/mL)	168.3 (186.5)	84.1 (99.8)	0.02
sCD23 (pg/mL)	5084 (5435)	4068 (3071)	<0.05
CD19^+^ CD11c^+^IgD^-^CD27^-^%	3.1 (3.7)	1.8 (1.4)	0.04
CD19^+^ CD11c^+^ %	10 (4.2)	6 (3.3)	0.07
sPD-1 (pg/mL)	123.5 (64)	88.3 (47.0)	0.06
sCD25 (pg/mL)	857.2 (1190.8)	2505 (3103)	0.09

Data displayed as median (interquartile range). Differences between responders (n=10) and nonresponders (n=13) were assessed by the Mann-Whitney test. P values < 0.05 considered significant.

### Immunologic markers associated with autoantibodies

Our data showed a strong correlation between anti-CCP and IgM RF levels (r=0.76, p<0.0001). To further identify the pathways related to autoantibodies in seropositive RA, we investigated potential links between multiple immunologic markers and anti-CCP and IgM RF (**Table 3**). We found that Tfh and Tph cell related markers (CXCL13, IL-21, sPD-1) were associated with anti-CCP or IgM RF. Of note, most of these markers including sPD-1 correlated more strongly with IgM RF compared to anti-CCP. Among T cell subsets, frequencies of CD4+CXCR5+PD-1hi Tfh cells and CD4+CXCR5-PD-1hi Tph cells were associated with both anti-CCP and Ig RF. Among B cell subsets, frequencies of CD19+IgD-CD27- B cell and CD19+CD11c+IgD-CD27- B cell were associated with anti-CCP but not with IgM RF. In Lasso regression, we found levels of anti-CCP and frequencies of CD19^+^CD11c^+^IgD^-^CD27^-^ B cells independently associated with the response to abatacept. Notably, the frequencies of CD19^+^CD11c^+^IgD^-^CD27^-^ B cells appeared to be more strongly associated with the response to abatacept as compared to anti-CCP, as indicated by their higher coefficient (**Supplementary Table 2**).

**Table 3:** Markers associated with anti-CCP and IgM RF in all RA patients (n=23).

Markers	r (anti-CCP)	p (anti-CCP)	r (IgM RF)	p (IgM RF)
CXCL13	0.59	0.002	0.66	0.001
sCD27	0.50	0.01	0.52	0.01
IL-21	0.49	0.01	0.66	<0.001
CXCL10	0.50	0.01	0.45	0.03
sPD-1	0.50	0.01	0.83	<0.001
CXCR5^+^PD-1^hi^CD4^+^ %	0.57	0.004	0.44	0.04
CXCR5^-^PD-1^hi^CD4^+^ %	0.58	0.004	0.42	<0.05
IgD^-^CD27^-^CD19^+^ %	0.51	0.014	0.30	0.17
CD11c^+^IgD^-^CD27^-^CD19^+^ %	0.42	<0.05	0.27	0.23
CD19^+^ IgD^-^CD27^-^%	0.51	0.01	0.30	ns
CD19^+^ CD11c^+^IgD^-^CD27^-^%	0.42	0.05	0.27	ns

Correlations of markers with anti-CCP and IgM RF at baseline assessed by Spearman’s correlation test. P values < 0.05 considered significant and ns considered non-significant.

### Shared epitope related to response to abatacept

**Figure 1:**
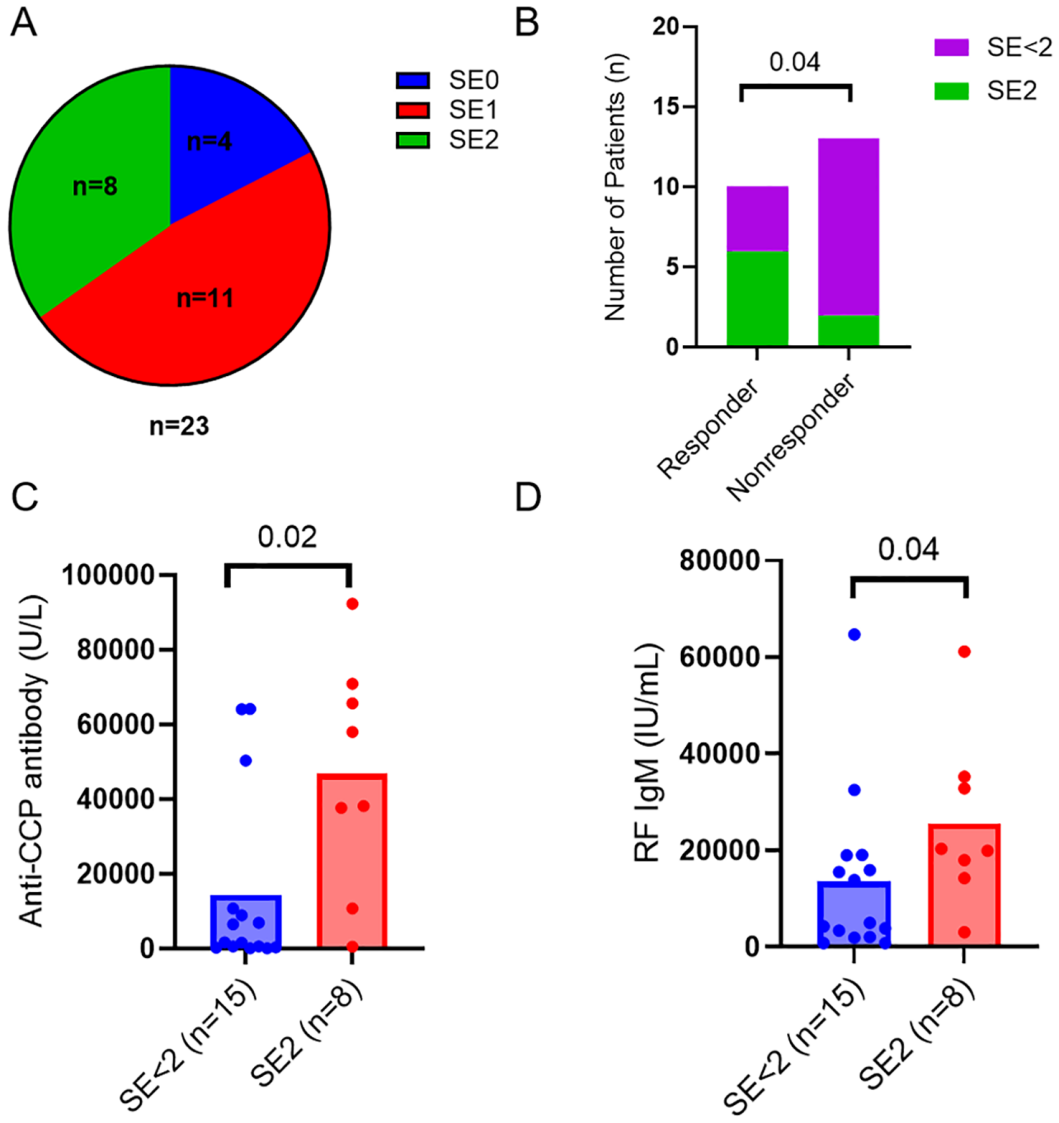
HLA-DRB1 shared epitope related to response to abatacept. **(A)** Pie chart showing numbers of RA patients with different SE alleles in the cohort. **(B)** Numbers of RA patients with two SE alleles (SE2) or less (SE<2) in responders and nonresponders. Differences between groups were determined by Fisher’s exact test. Baseline serum levels of anti-CCP antibodies **(C)** and RF IgM **(D)** in the RA patients with 2 alleles of shared epitope (SE2) and the control patients with 1 or no SE allele (SE<2). Differences between groups were determined by the Mann-Whitney U test. P values < 0.05 were considered significant.

Besides immunologic markers, RA patients were also genotyped for HLA-DR SE alleles and classified as SE2 (≥ 2 SE allele, n=8), SE1 (1 SE allele, n=11), and SE0 (no SE alleles, n=4) (**Figure 1A**). The presence of two alleles of shared epitope (SE) was associated with ACR50 response (p=0.04, **Figure 1B**) and was not significantly associated with ACR20 response (p=0.09, data not shown). Patients with 2 alleles of shared epitope had higher levels of anti-CCP (p=0.02, **Figure 1C**) and IgM RF (p=0.04, **Figure 1D**) when compared to patients with 1 or no SE allele.

### Longitudinal changes in T cell and B cell related soluble and cellular markers

**Figure 2:**
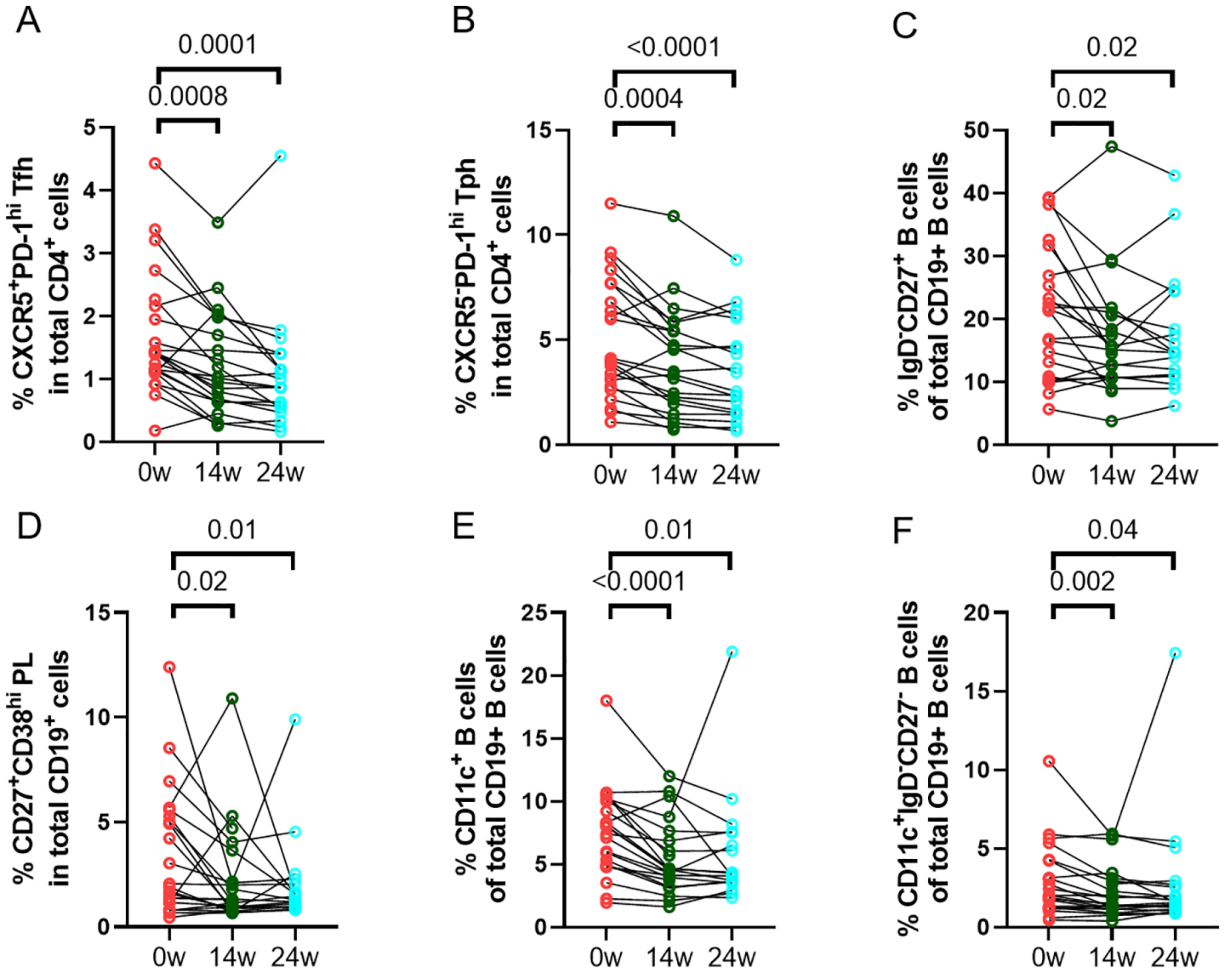
Changes in percentages of immune cell phenotypes. Frequencies of CD4^+^CXCR5^+^PD-1^hi^ Tfh cells **(A)**, CD4^+^CXCR5^-^PD-1^hi^ Tph cells **(B)**, CD19^+^IgD^-^CD27^+^ class-switched memory (SM) B cells **(C)**, CD19^+^CD27^+^CD38^hi^ plasmablasts/plasma (PL) B cells**(D)**, CD19^+^CD11c^+^ B cells **(E)**, and CD19^+^CD11c^+^IgD^-^CD27^-^ B cells **(F)** in all RA patients (n=23) were documented before and after treatment with abatacept. Differences between baseline and post-treatment (week 14 and week 24) were determined by the Wilcoxon matched-pairs signed rank test. P values < 0.05 were considered significant.

We assessed the changes of frequencies of different immune cell phenotypes and levels of soluble markers to determine the effect of abatacept treatment on these markers. There was a significant decrease in frequencies of circulating CD4^+^CXCR5^+^PD-1^hi^ Tfh cells (**Figure 2A**) and CD4^+^CXCR5^-^PD-1^hi^ Tph cells (**Figure 2B**) at week 14 and week 24. There was also a significant reduction in other T cell subsets, including CD4^+^CD25^+^CD127^-^ regulatory T (Treg) cells, CD4^+^CD45RA^-^CCR7^+^ central memory T cells, and CD4^+^CD45RA^-^CCR7^-^ effector memory T cells (**Supplementary Table 3**). Furthermore, abatacept decreased ICOS expression on both Tfh cells and Tph cells at week 14 (p<0.0001 and p<0.0001, respectively) and week 24 (p<0.0001 and p<0.0001, respectively) (**Supplementary Figure 3**). Abatacept also decreased frequencies of circulating CD19^+^IgD^-^CD27^+^ class-switched memory (SM) B cells, CD19^+^CD27^+^CD38^hi^ plasmablasts/plasma (PL) B cells, CD19^+^CD11c^+^ B cells and CD19^+^CD11c^+^IgD^-^CD27^-^ B cells at week 14 and week 24 (**Figures 2C–F**; **Supplementary Table 4**) as well as CD11c expression on B cells at week 14 and 24 (p=0.01 and p=0.006 respectively) (**Supplementary Figure 4**). Serum levels of sCD23, CXCL13 and RF IgM were significantly decreased at week 14 and week 24 with the treatment of abatacept when compared to baseline, whiles sCD27, CD40L and IL-21 levels were only decreased at week 14, sCD25, and sPD-1 were only reduced at week 24 (**Table 4**). Changes of anti-CCP levels did not reach statistical significance (**Table 4**).

**Table 4:** Levels of soluble markers before and after treatment with abatacept in all RA patients (n=23).

Markers	Baseline	Week 14	Week 24
sCD23 (pg/mL)	4244 (3205)	3733 (3124) ^****^	4009 (2603) ^**^
sCD25 (pg/mL)	1368 (2845.6)	909.7 (1626.15) ^****^	723.3 (1652.3) ^**^
sCD27 (pg/mL)	177 (342.79)	135.7 (198.11) ^**^	97.08 (185.88) ^****^
sCD40L (pg/mL)	18.17 (22.329)	19.18 (17.18) ^**^	15.74 (22.842)
CXCL13 (pg/mL)	119.1 (188.06)	85.27 (83) ^***^	67.91 (57.83) ^**^
IL-21 (pg/mL)	39.06 (125.8)	12.48 (54.47) ^**^	0 (45.46)
sPD-1 (pg/mL)	95.21 (64.05)	83.46 (31.94) ^****^	81.03 (34.5) ^*^
RF IgM (IU/mL)	15894 (16520)	13278 (18237) ^**^	12122 (19298) ^*^
Anti-CCP (U/mL)	10799 (53629)	10624 (50937.4)	9381 (44051.6)

Data are displayed as median (interquartile range). Differences between baseline and post-treatment (week 14 and week 24) were assessed by the Wilcoxon matched-pairs signed rank test. ****p<0.0001, ***p<0.001, **p<0.01 and *p<0.05.

### CXCL13 and IgM RF as pharmacodynamic biomarkers for abatacept treatment

**Figure 3:**
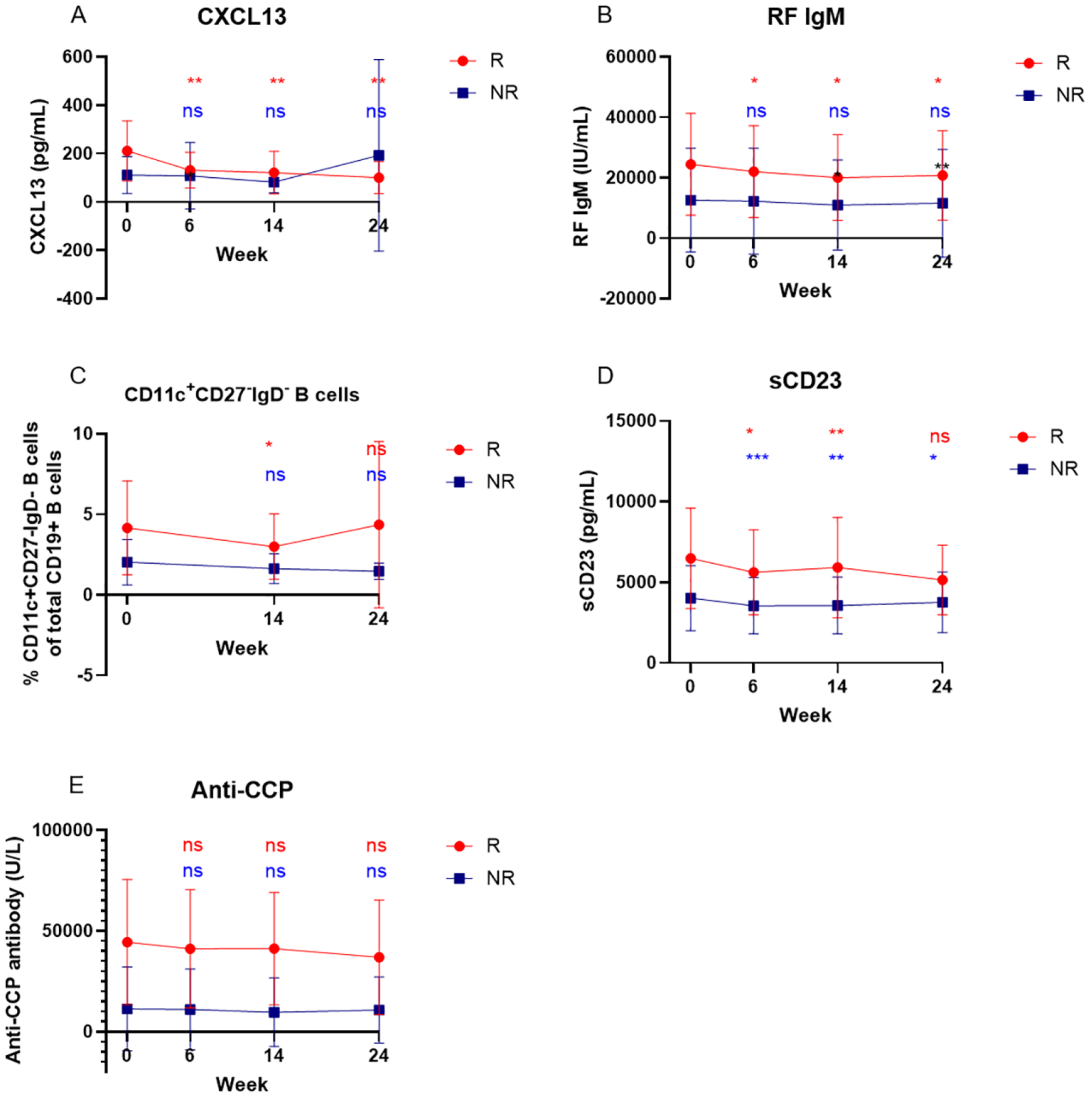
Serum CXCL13, RF IgM but not anti-CCP levels decreased in responders with treatment of abatacept. Data are displayed as a mean of serum levels of CXCL13 **(A)**, RF IgM **(B)**, CD11c_+_CD27_-_IgD_-_ B cells **(C)**, sCD23 **(D)**, and anti-CCP antibodies **(E)** before and after treatment of abatacept in responders (n=10) and non-responders (n=13), error bars display the 95% confidence. Differences between baseline and post-treatment (week 6, week 14, and week 24) were determined by the Wilcoxon matched-pairs signed rank test. P values < 0.05 were considered significant. **p<0.01, *p<0.05, ***p<0.001 and ns considered non-significant.

We compared the longitudinal changes in T and B cell related markers including IgM RF and anti-CCP levels between responders and nonresponders. There were differences in longitudinal alterations in CXCL 13 (**Figure 3A**) and IgM RF (**Figure 3B**) levels between responders and nonresponders. CXCL13 and IgM RF levels decreased only in responders who achieved either ACR50 response (p=0.004 and p=0.02, respectively, at week 24) or ACR20 (p=0.03 and p=0.006, respectively, at week 24, data not shown). Frequencies of CD19^+^CD11c^+^IgD^-^CD27^-^ B cells only decreased in responders at week 14 (p=0.02) but not at week 24 (**Figure 3C**). On the other hand, sCD23 (**Figure 3D**) were reduced significantly in both responders and nonresponders, while anti-CCP levels (**Figure 3E**) did not decrease significantly in either responders or nonresponders.

## Discussion

In this study, we have reported several immune markers which are associated with the response to abatacept in RA patients. Though majority of patients had some response with abatacept, we chose the ACR50 response at week 24 as the responder criteria to identify the markers that are associated with a major improvement in disease activity from abatacept therapy. Consistent with previous studies (5, 6), abatacept was more effective in seropositive rheumatoid arthritis (RA) patients with positive anti-CCP and RF.

There have been new approaches to try to predict the response to biologic agents based on synovial biopsy phenotype in RA (1, 21). However synovial biopsy is not available nor standardized in most of rheumatology clinics currently and peripheral blood markers would be preferred by many clinicians, not least due to blood sampling being less invasive. In this study, T and B lymphocyte related markers including CXCL13, IL-21, sPD-1 and sCD23 were significantly decreased with the treatment of abatacept. CXCL13, IL-21 and PD-1 are expressed by T follicular helper (Tfh) cells and T peripheral helper (Tph) cells infiltrated in RA synovium (3) which play crucial roles in stimulating B cell activation and autoantibody production.

Similar to our findings, levels of CXCL13 and sPD-1 levels have been shown to correlate with RF titers in RA patients (19, 22). CXCL13 is involved in recruiting CXCR5+ B cells to synovium in RA and was associated with disease activity (23), ultrasonographic synovitis (15) and long-term radiographic progression (24). CXCL13 is mainly secreted by follicular dendritic cells and Tfh cells (25) and abatacept decreases its levels by suppressing activation of these two cells through interrupting the interaction between CD80/86 and CD28. Additionally, high CXCL13 levels were observed in RA patients with the lymphoid phenotype in synovium (1). These patients responded better to abatacept as shown in our study. Thus, though validation is required, CXCL13, reflecting T- and B-lymphocyte-driven synovial inflammation, may be a novel blood-based marker of treatment response to abatacept.

Our data showed that baseline frequencies of CD19^+^CD11c^+^IgD^-^CD27^-^ B cells were higher in responders to abatacept. These cells are commonly referred to as double-negative (DN) 2 B cells (11) and have been identified in contexts of infection (26) and autoimmune diseases (27), where they are often termed atypical memory B cells or age-associated B cells (ABCs), as characterized by the expression of the T-box transcription factor (T-bet) (28, 29). It’s been revealed that CD11c^hi^CD27^low^T-bet^+^ B cells are primed to differentiate into plasma cells and produce autoantibodies targeting RNA-associated antigens such as Sm and SmD in SLE (30). In RA, DN2 B cells are major precursors to synovial antibody-secreting cells (31). To our knowledge, this is the first study to show an association between CD19^+^CD11c^+^IgD^-^CD27^-^ B cells and response to abatacept in RA. Additionally, we found that CD11c levels on B cells decreased with abatacept treatment. Tfh cells have been reported as a major source of IL-21 in various inflammatory and autoimmune diseases (32–34). In alignment with these findings, our data also showed that abatacept treatment significantly reduced serum IL-21 levels in RA patients. It’s been reported that IL-21 can drive the expansion of CD11c^hi^T-bet^+^ B cells (DN2) and promote their differentiation into Ig-producing autoreactive plasma cells (30) and that IL-21 and interferon-γ (IFNγ)-coexpressing PD-1^high^ CXCR5^-^HLA-DR^+^ Tph cells skewed B cell differentiation toward a CD21^low^ CD11c^+^ phenotype *in vitro* (35). Based on these findings, we hypothesized that CD28 blockade by abatacept disrupts the development and maintenance of the Tfh and Tph cells, thereby suppressing DN2 B cell expansion through decreased IL-21 production.

The percentage of B cells expressing CD23, the low-affinity receptor for the Fc portion of IgE, and serum levels of soluble CD23 (sCD23) were increased in patients with RA (16). Soluble CD23 is cleaved from naïve B cells when these cells are matured to memory B cells and the levels of sCD23 decreased with B cell depletion therapy, rituximab, in RA patients (36). We have shown abatacept was more effective in RA patients with a higher sCD23 levels which reflected B cell activation and differentiation. Our findings are thus consistent with the known mechanism of action of abatacept, inhibiting Tfh and Tph cell activation that leads to suppression of B cell differentiation.

On the other hand, soluble CD25 (sCD25) levels were numerically higher in nonresponders. Soluble CD25 is a high-affinity soluble IL-2 receptor and can form a complex with IL-2. This cytokine-receptor complex can potentially activate various cells including CD4 and CD8 effector T cells and NK cells through “trans presentation” of IL-2 to the cells (17). We found that sCD25 levels were lower in RA patients with 2 alleles of shared epitope (data not shown). Based on those observations, sCD25 may be associated with a distinct pathway of immune cell activation for seronegative RA that is not responsive to B cell modulation by abatacept.

Furthermore, RA patients with two shared epitope (SE) alleles had a higher probability to achieve ACR50 response from abatacept therapy and there seemed a close relationship between presence of shared epitopes and anti-CCP levels. Though a previous study showed abatacept resulted in higher efficacy responses versus adalimumab after 24 weeks in SE-positive patients (6), this is the first study to demonstrate two SE alleles being associated with treatment response to abatacept. This genetic marker will add additional information in evaluating RA if used along with anti-CCP levels as the latter may change with treatment.

Lastly, we found CXCL13 and IgM RF levels decreased with treatment only in responders to abatacept whereas anti-CCP levels did not decrease in either responders or nonresponders. Abatacept would inhibit production of autoantibodies including RF by suppressing Tfh and Tph cells. Given that we measure IgM isotype of RF which are secreted by newly activated B cells, IgM RF levels would be more preferably decreased by abatacept compared to IgG autoantibodies. Our results were consistent with the report that rheumatoid factor levels correlated with disease activity better than anti-CCP in RA patients (37). We plan to conduct an additional analysis of RA clinical trial data to determine if these markers could be used to monitor the response to abatacept.

The limitations of this study include subject number and study population. First and foremost, our study had a small number of subjects recruited from a single center and our results should be validated in a larger RA cohort. It would be useful to assess these markers in a specific RA population including early-onset RA or anti-CCP high RA patients. Additionally, our study population was primarily patients who had an inadequate response to DMARDs and biologic-naïve. It would be interesting to evaluate if these markers are also associated with the response to abatacept in patients who had inadequate response to biologic agents including TNF inhibitors. The exact function of DN2 B cells in RA, their interaction with T cells, and their BCR repertoire, are beyond the scope of this current study. However, there is a significant interest in this B cell subset in RA which should be further evaluated in the future studies.

In conclusion, we have shown the markers of B cell activation including frequencies of CD19^+^CD11c^+^IgD^-^CD27^-^ B cells in RA were associated with favorable response to abatacept. Further, RA patients with two copies of shared epitope had a higher probability to achieve ACR 50 response. Though multiple markers related to immune cell activation decreased with abatacept therapy, CXCL13 and IgM RF decreased only in responders and could be potentially used as pharmacodynamic markers for abatacept in RA treatment.

## Data Availability

All relevant data is contained within the article: The original contributions presented in the study are included in the article/**Supplementary Material**, further inquiries can be directed to the corresponding author.

## References

[1] Dennis G Holweg CTJ Kummerfeld SK Choy DF Setiadi AF Hackney JA. Synovial phenotypes in rheumatoid arthritis correlate with response to biologic therapeutics. Arthritis Res Ther. (2014) 16:R90. doi: 10.1186/ar4555 25167216 PMC4060385

[2] Orr C Najm A Biniecka M McGarry T Ng CT Young F. Synovial immunophenotype and anti-citrullinated peptide antibodies in rheumatoid arthritis patients: relationship to treatment response and radiologic prognosis. Arthritis Rheumatol. (2017) 69:2114–23. doi: 10.1002/art.v69.11 28732135

[3] Zhang F Wei K Slowikowski K Fonseka CY Rao DA Kelly S. Defining inflammatory cell states in rheumatoid arthritis joint synovial tissues by integrating single-cell transcriptomics and mass cytometry. Nat Immunol. (2019) 20:928–42. doi: 10.1038/s41590-019-0378-1 PMC6602051 31061532

[4] Rao DA Gurish MF Marshall JL Slowikowski K Fonseka CY Liu Y. Pathologically expanded peripheral T helper cell subset drives B cells in rheumatoid arthritis. Nature. (2017) 542:110–4. doi: 10.1038/nature20810 PMC5349321 28150777

[5] Sokolove J Schiff M Fleischmann R Weinblatt ME Connolly SE Johnsen A. Impact of baseline anti-cyclic citrullinated peptide-2 antibody concentration on efficacy outcomes following treatment with subcutaneous abatacept or adalimumab: 2-year results from the AMPLE trial. Ann Rheum Dis. (2016) 75:709–14. doi: 10.1136/annrheumdis-2015-207942 PMC4819608 26359449

[6] Rigby W Buckner JH Louis Bridges S Nys M Gao S Polinsky M. HLA-DRB1 risk alleles for RA are associated with differential clinical responsiveness to abatacept and adalimumab: data from a head-to-head, randomized, single-blind study in autoantibody-positive early RA. Arthritis Res Ther. (2021) 23:245. doi: 10.1186/s13075-021-02607-7 34537057 PMC8449494

[7] Platt AM Gibson VB Patakas A Benson RA Nadler SG Brewer JM. Abatacept limits breach of self-tolerance in a murine model of arthritis via effects on the generation of T follicular helper cells. J Immunol. (2010) 185:1558–67. doi: 10.4049/jimmunol.1001311 20601593

[8] Verstappen GM Meiners PM Corneth OBJ Visser A Arends S Abdulahad WH. Attenuation of follicular helper T cell-dependent B cell hyperactivity by abatacept treatment in primary sjögren’s syndrome. Arthritis Rheumatol. (2017) 69:1850–61. doi: 10.1002/art.40165 28564491

[9] Glatigny S Höllbacher B Motley SJ Tan C Hundhausen C Buckner JH. Abatacept targets T follicular helper and regulatory T cells, disrupting molecular pathways that regulate their proliferation and maintenance. J Immunol. (2019) 202:1373–82. doi: 10.4049/jimmunol.1801425 PMC6481683 30683697

[10] Scarsi M Paolini L Ricotta D Pedrini A Piantoni S Caimi L. Abatacept reduces levels of switched memory B cells, autoantibodies, and immunoglobulins in patients with rheumatoid arthritis. J Rheumatol. (2014) 41:666–72. doi: 10.3899/jrheum.130905 24584924

[11] Jenks SA Cashman KS Zumaquero E Marigorta UM Patel AV Wang X. Distinct effector B cells induced by unregulated toll-like receptor 7 contribute to pathogenic responses in systemic lupus erythematosus. Immunity. (2018) 49:725–39. doi: 10.1016/j.immuni.2018.08.015 PMC6217820 30314758

[12] Sanz I Wei C Jenks SA Cashman KS Tipton C Woodruff MC. Challenges and opportunities for consistent classification of human B cell and plasma cell populations. Front Immunol. (2019) 10:2458/full. doi: 10.3389/fimmu.2019.02458/full 31681331 PMC6813733

[13] Rubtsov AV Rubtsova K Kappler JW Jacobelli J Friedman RS Marrack P. CD11c-expressing B cells are located at the T cell B cell border in spleen and are potent antigen presenting cells. J Immunol. (2015) 195:71–9. doi: 10.4049/jimmunol.1500055 PMC4475418 26034175

[14] Crotty S. T follicular helper cell differentiation, function, and roles in disease. Immunity. (2014) 41:529–42. doi: 10.1016/j.immuni.2014.10.004 PMC4223692 25367570

[15] Bugatti S Manzo A Benaglio F Klersy C Vitolo B Todoerti M. Serum levels of CXCL13 are associated with ultrasonographic synovitis and predict power Doppler persistence in early rheumatoid arthritis treated with non-biological disease-modifying anti-rheumatic drugs. Arthritis Res Ther. (2012) 14:R34. doi: 10.1186/ar3742 22336440 PMC3392832

[16] Chomarat P Briolay J Banchereau J Miossec P. Increased production of soluble CD23 in rheumatoid arthritis, and its regulation by interleukin-4. Arthritis Rheumatol. (1993) 36:234–42. doi: 10.1002/art.1780360215 8431213

[17] Damoiseaux J. The IL-2 - IL-2 receptor pathway in health and disease: The role of the soluble IL-2 receptor. Clin Immunol. (2020) :218:108515. doi: 10.1016/j.clim.2020.108515 32619646

[18] Shoda H Nagafuchi Y Tsuchida Y Sakurai K Sumitomo S Fujio K. Increased serum concentrations of IL-1 beta, IL-21 and Th17 cells in overweight patients with rheumatoid arthritis. Arthritis Res Ther. (2017) 19:111. doi: 10.1186/s13075-017-1308-y 28569167 PMC5452609

[19] Jones JD Hamilton BJ Challener GJ de Brum-Fernandes AJ Cossette P Liang P. Serum C-X-C motif chemokine 13 is elevated in early and established rheumatoid arthritis and correlates with rheumatoid factor levels. Arthritis Res Ther. (2014) 16:R103. doi: 10.1186/ar4552 24766912 PMC4060390

[20] Aletaha D Neogi T Silman AJ Funovits J Felson DT Bingham CO. 2010 Rheumatoid arthritis classification criteria: an American College of Rheumatology/European League Against Rheumatism collaborative initiative. Arthritis Rheum. (2010) 62:2569–81. doi: 10.1002/art.27584 20872595

[21] Zhang F Jonsson AH Nathan A Millard N Curtis M Xiao Q. Deconstruction of rheumatoid arthritis synovium defines inflammatory subtypes. Nature. (2023) 623:616–24. doi: 10.1038/s41586-023-06708-y PMC10651487 37938773

[22] Wan B Nie H Liu A Feng G He D Xu R. Aberrant regulation of synovial T cell activation by soluble costimulatory molecules in rheumatoid arthritis. J Immunol. (2006) 177:8844–50. doi: 10.4049/jimmunol.177.12.8844 17142787

[23] Rioja I Hughes FJ Sharp CH Warnock LC Montgomery DS Akil M. Potential novel biomarkers of disease activity in rheumatoid arthritis patients: CXCL13, CCL23, transforming growth factor alpha, tumor necrosis factor receptor superfamily member 9, and macrophage colony-stimulating factor. Arthritis Rheumatol. (2008) 58:2257–67. doi: 10.1002/art.23667 18668547

[24] Greisen SR Mikkelsen C Hetland ML Østergaard M Hørslev-Petersen K Junker P. CXCL13 predicts long-term radiographic status in early rheumatoid arthritis. Rheumatol (Oxford). (2022) 61:2590–5. doi: 10.1093/rheumatology/keab763 34636880

[25] Pan Z Zhu T Liu Y Zhang N. Role of the CXCL13/CXCR5 axis in autoimmune diseases. Front Immunol. (2022) 13:850998. doi: 10.3389/fimmu.2022.850998 35309354 PMC8931035

[26] Weiss GE Crompton PD Li S Walsh LA Moir S Traore B. Atypical memory B cells are greatly expanded in individuals living in a malaria-endemic area. J Immunol. (2009) 183:2176–82. doi: 10.4049/jimmunol.0901297 PMC2713793 19592645

[27] Claes N Fraussen J Vanheusden M Hellings N Stinissen P Van Wijmeersch B. Age-associated B cells with proinflammatory characteristics are expanded in a proportion of multiple sclerosis patients. J Immunol. (2016) 197:4576–83. doi: 10.4049/jimmunol.1502448 27837111

[28] Naradikian MS Myles A Beiting DP Roberts KJ Dawson L Herati RS. Cutting edge: IL-4, IL-21, and IFN-γ Interact to govern T-bet and CD11c expression in TLR-activated B cells. J Immunol. (2016) 197:1023–8. doi: 10.4049/jimmunol.1600522 PMC4975960 27430719

[29] Rubtsova K Rubtsov AV van Dyk LF Kappler JW Marrack P. T-box transcription factor T-bet, a key player in a unique type of B-cell activation essential for effective viral clearance. Proc Natl Acad Sci. (2013) 110:E3216–24. doi: 10.1073/pnas.1312348110 PMC3752276 23922396

[30] Wang S Wang J Kumar V Karnell JL Naiman B Gross PS. IL-21 drives expansion and plasma cell differentiation of autoreactive CD11chiT-bet+ B cells in SLE. Nat Commun. (2018) 9:1758. doi: 10.1038/s41467-018-03750-7 29717110 PMC5931508

[31] Wing E Sutherland C Miles K Gray D Goodyear CS Otto TD. Double-negative-2 B cells are the major synovial plasma cell precursor in rheumatoid arthritis. Front Immunol. (2023) 14:1241474/full. doi: 10.3389/fimmu.2023.1241474/full 37638026 PMC10450142

[32] Gbedande K Domingo NN Puebla-Clark L Weinstein JD Stephens R. IL-21 from IFN-g +IL-21 +hybrid T cells promotes germinal center B cell proliferation. J Immunol. (2023) 210:241.03. doi: 10.4049/jimmunol.210.Supp.241.03

[33] Pontarini E Murray-Brown WJ Croia C Lucchesi D Conway J Rivellese F. Unique expansion of IL-21+ Tfh and Tph cells under control of ICOS identifies Sjögren’s syndrome with ectopic germinal centres and MALT lymphoma. Ann Rheum Dis. (2020) 79:1588–99. doi: 10.1136/annrheumdis-2020-217646 PMC7677495 32963045

[34] Zander R Kasmani MY Chen Y Topchyan P Shen J Zheng S. Tfh-cell-derived interleukin 21 sustains effector CD8+ T cell responses during chronic viral infection. Immunity. (2022) 55:475–93. doi: 10.1016/j.immuni.2022.01.018 PMC8916994 35216666

[35] Fischer J Dirks J Klaussner J Haase G Holl-Wieden A Hofmann C. Effect of clonally expanded PD-1high CXCR5-CD4+ Peripheral T helper cells on B cell differentiation in the joints of patients with antinuclear antibody-positive juvenile idiopathic arthritis. Arthritis Rheumatol. (2022) 74:150–62. doi: 10.1002/art.41913 34196496

[36] Cambridge G Perry HC Nogueira L Serre G Parsons HM de la Torre I. The effect of B-cell depletion therapy on serological evidence of B-cell and plasmablast activation in patients with rheumatoid arthritis over multiple cycles of rituximab treatment. J Autoimmun. (2014) 50:67–76. doi: 10.1016/j.jaut.2013.12.002 24365380

[37] Aletaha D Alasti F Smolen JS. Rheumatoid factor, not antibodies against citrullinated proteins, is associated with baseline disease activity in rheumatoid arthritis clinical trials. Arthritis Res Ther. (2015) 17:229. doi: 10.1186/s13075-015-0736-9 26307354 PMC4549866

