# Supplementary material for: Evaluation of B cell related markers and autoantibodies in rheumatoid arthritis patients treated with abatacept
Source: Front Immunol. 2025 Jan 24;16:1504454. doi: 10.3389/fimmu.2025.1504454 (PMC11803405; doi:10.3389/fimmu.2025.1504454)
Supplement: Supplementary file 1 [file DataSheet1.docx]

**Supplementary material**

**Supplementary Table 1. Antibodies used for flow cytometry.**

| Antibodies | Clone | Company |
| --- | --- | --- |
| CD3 | UCHT1 | Beckman Coulter |
| CD3 | HIT3a | BioLegend |
| CD4 | OKT4 | BioLegend |
| CD8 | SK1 | BioLegend |
| CD11c | 3.9 | Invitrogen |
| CD19 | HIB19 | BioLegend |
| CD25 | BC96 | BioLegend |
| CD27 | M-T271 | BioLegend |
| CD28 | CD28.2 | BioLegend |
| CD38 | HB-7 | BioLegend |
| CD45RA | HI100 | BioLegend |
| CD127 | A019D5 | BioLegend |
| CXCR5 | J252D4 | BioLegend |
| PD-1 | EH12.2H7 | BioLegend |
| ICOS | C398.4A | BioLegend |
| CCR7 | G043H7 | BioLegend |
| IgD | IA6-2 | BioLegend |

**Supplementary Table 2.** Lasso regression to show the association between different markers with response to abatacept. The coefficients represent the estimated effect of each predictor variable on the response variable. The sign of the coefficient indicates the direction of the relationship (positive means as the independent variable increases, so does the dependent variable, while negative means the opposite). The absolute value of the coefficient indicates the strength of the relationship; a larger coefficient means a bigger impact on the dependent variable for a unit change in the independent variable. “.” indicates no relationship between the predictor and the response.

| Variables | Coefficients |
| --- | --- |
| Anti-CCP (U/mL) | 7.928044e-06 |
| RF IgM (IU/mL) | . |
| CXCL13 (pg/mL) | . |
| sCD23 (pg/mL) | . |
| CD11c^+^IgD^-^CD27^-^CD19^+^ % | 1.639747e-02 |

**Supplementary Table 3.** Changes in frequencies of T cell subsets. Data are displayed as median (interquartile range). Differences between baseline and post-treatment (week 14 and week 24) were assessed by the Wilcoxon matched-pairs signed rank test. ****P<0.0001, ***P<0.001, **P<0.01 and *P<0.05.

| T cell subsets | Baseline | Week 14 | Week 24 |
| --- | --- | --- | --- |
| **CD4+ T cell subsets** |  |  |  |
| Follicular helper T  (Tfh, CD4^+^CXCR5^+^PD-1^hi^) | 1.4 (1.0) | 1.0 (1.3) ^***^ | 0.9 (0.8) ^***^ |
| Peripheral helper T  (Tph, CD4^+^CXCR5^-^PD-1^hi^) | 4.0 (4.9) | 3.5 (3.5) ^***^ | 3.0 (4.2) ^****^ |
| Treg  (CD4^+^CD25^+^CD127^-^) | 5.9 (2.3) | 4.2 (1.71) ^****^ | 4.2 (1.8) ^****^ |
| Naïve  (CD4^+^CD45RA^+^CCR7^+^) | 40.9 (19.5) | 45.5 (22.2) ^*^ | 41.2 (22.6) ^**^ |
| Central memory  (CD4^+^CD45RA^-^CCR7^+^) | 45.3 (11.3) | 46.0 (13.7) | 43.5 (12.8) ^*^ |
| Effector memory  (CD4^+^CD45RA^-^CCR7^-^) | 7.7 (8.2) | 6.6 (7.7) ^**^ | 7.2 (6.9) |
| **CD8+ T cell subsets** |  |  |  |
| Naïve  (CD8^+^CD45RA^hi^CCR7^+^) | 19.0 (15.5) | 20.5 (22.3) | 21.7 (14.4) |
| Transitional central memory (CD8^+^CD45RA^lo-int^ CCR7^+^) | 10.8 (5.0) | 11.8 (5.4) | 11.4 (6.5) |
| Central memory  (CD8^+^CD45RA^-^CCR7^+^) | 9.2 (7.1) | 7.5 (8.5) | 9.5 (5.9) |
| Effector memory  (CD8^+^CD45RA^-^CCR7^-^) | 9.3 (7.1) | 8.3 (8.2) | 9.4 (9.3) |
| Late effector memory  (CD8^+^CD45RA^lo-int^CCR7^-^) | 16.7 (13.9) | 15.6 (14.4) | 17.1 (12.8) |
| Terminally differentiated effector memory (CD8^+^CD45RA^hi^CCR7^-^) | 17.5 (19.6) | 17.3 (18.2) | 19.5 (22.6) |

**Supplementary Table 4.** Changes in frequencies of B cell subsets. Data are displayed as median (interquartile range). Differences between baseline and post-treatment (week 14 and week 24) were assessed by the Wilcoxon matched-pairs signed rank test. ****P<0.0001, ***P<0.001, **P<0.01 and *P<0.05.

| B cells subsets | Baseline | Week 14 | Week 24 |
| --- | --- | --- | --- |
| Naïve  (CD19^+^IgD^+^CD27^-^) | 54.0 (19.6) | 62.7 (18.1) ^*^ | 60.7 (18.3) |
| Class-switched memory  (SM, CD19^+^IgD^-^CD27^+^) | 21.4 (17.3) | 15.4 (10.1) ^*^ | 14.7 (13.3) ^*^ |
| Non-switched memory  (Non-SM, CD19^+^IgD^+^CD27^+^) | 10.6 (7.0) | 10.5 (8.7) | 10.8 (9.5) |
| Double negative  (DN, CD19^+^IgD^-^CD27^-^) | 9.1 (9.0) | 9.0 (7.0) | 9.4 (6.4) |
| Plasmablast  (PL, CD19^+^CD27^+^CD38^hi^) | 1.9 (4.1) | 1.2 (1.3) ^*^ | 1.4 (1.1) ^*^ |
| CD11c^+^  (CD19^+^CD11c^+^) | 7.7 (5.1) | 4.6 (3.5) ^****^ | 4.2 (4.0) ^*^ |
| CD11c^+^IgD^-^CD27^-^  (CD19^+^ CD11c^+^IgD^-^CD27^-^) | 2.0 (3.0) | 1.7 (1.8) ^**^ | 1.6 (1.5) ^*^ |


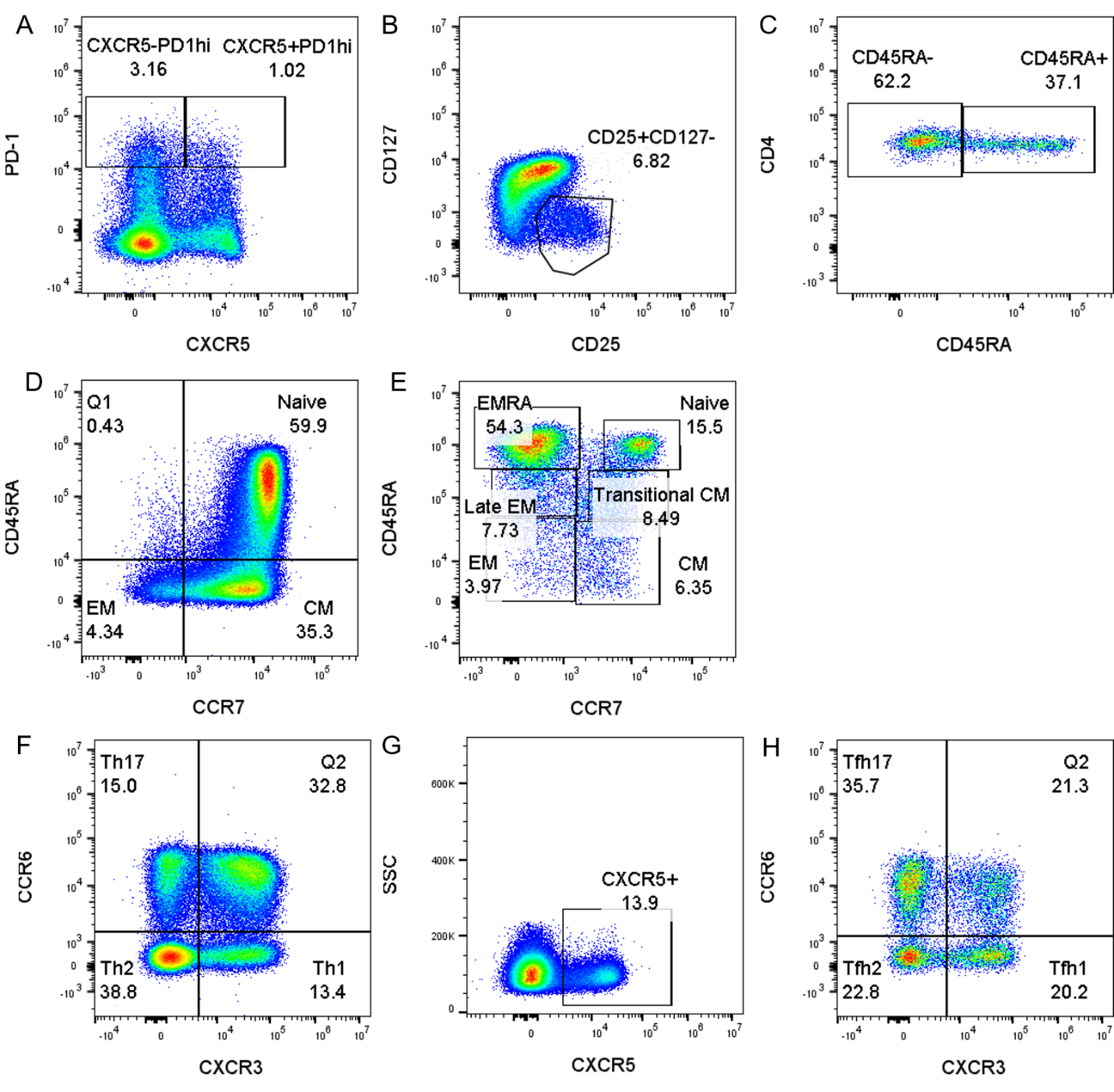

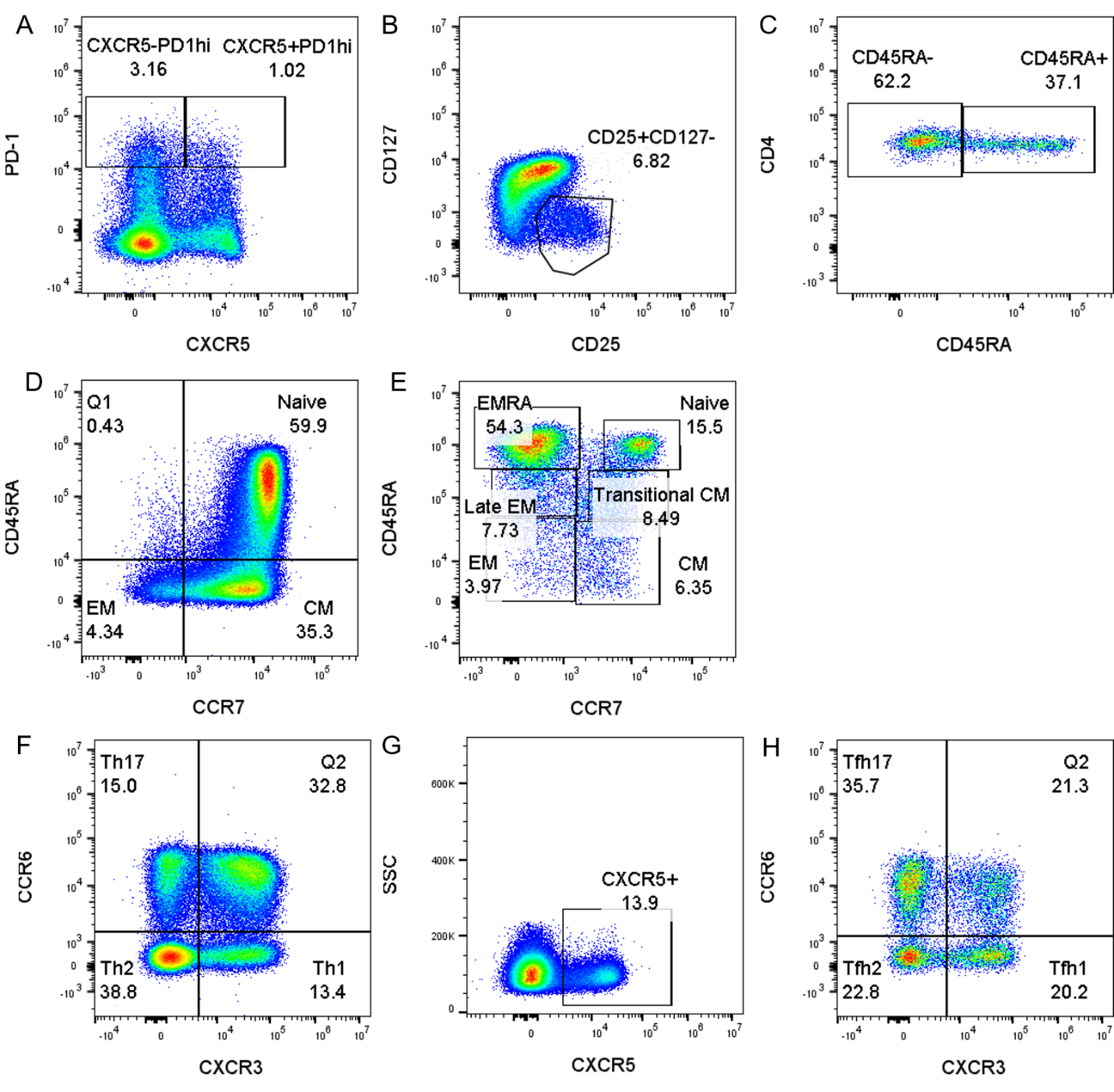

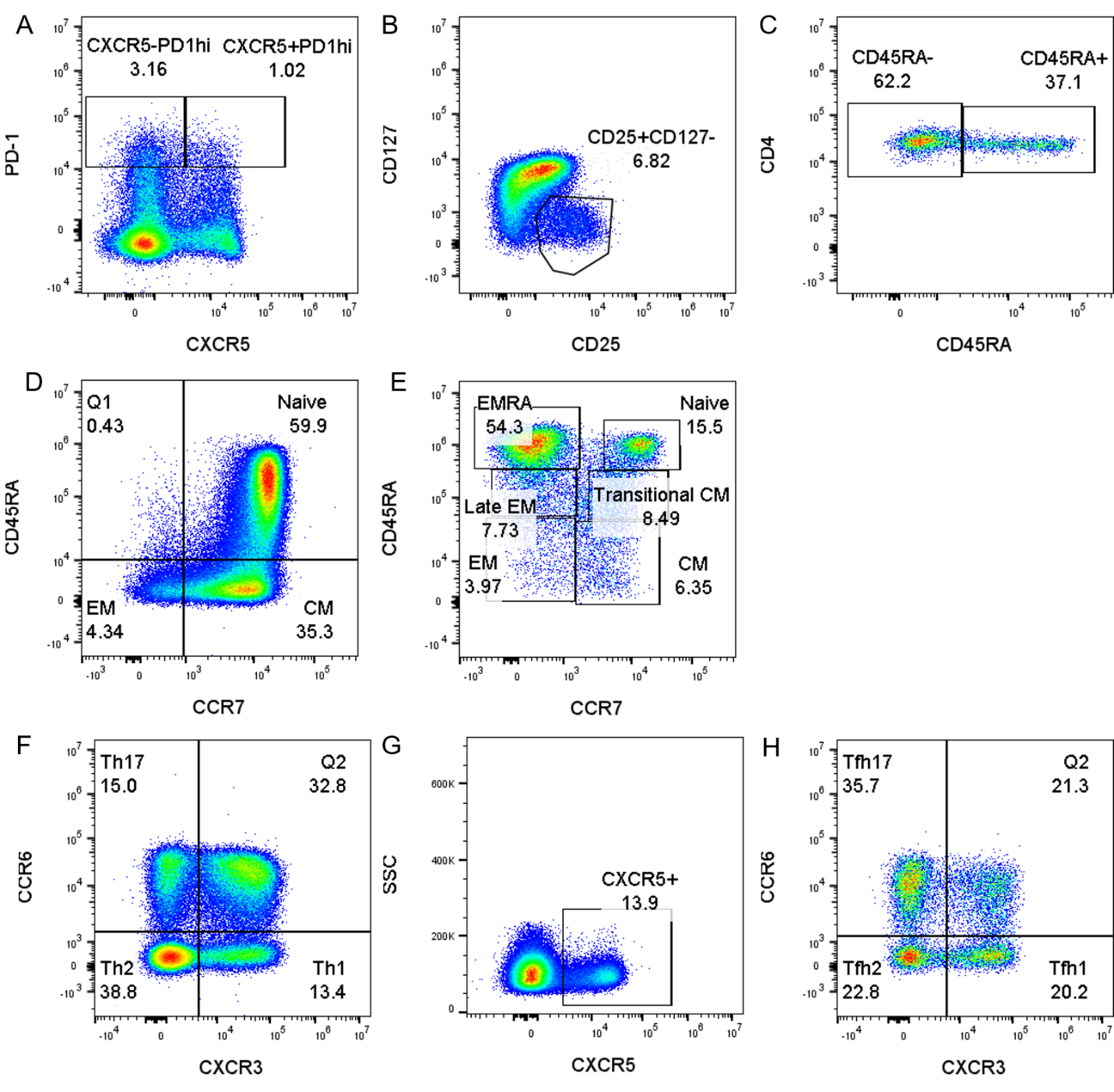


**Supplementary Figure 1: T cell phenotypes in patients with RA.** (A-C) Representative dot plots of T cell phenotypes on gated CD3+CD4+ T cells in patients with RA. (D) Representative gating strategies for CD3+CD8+ T cell subsets in patients with RA.

**
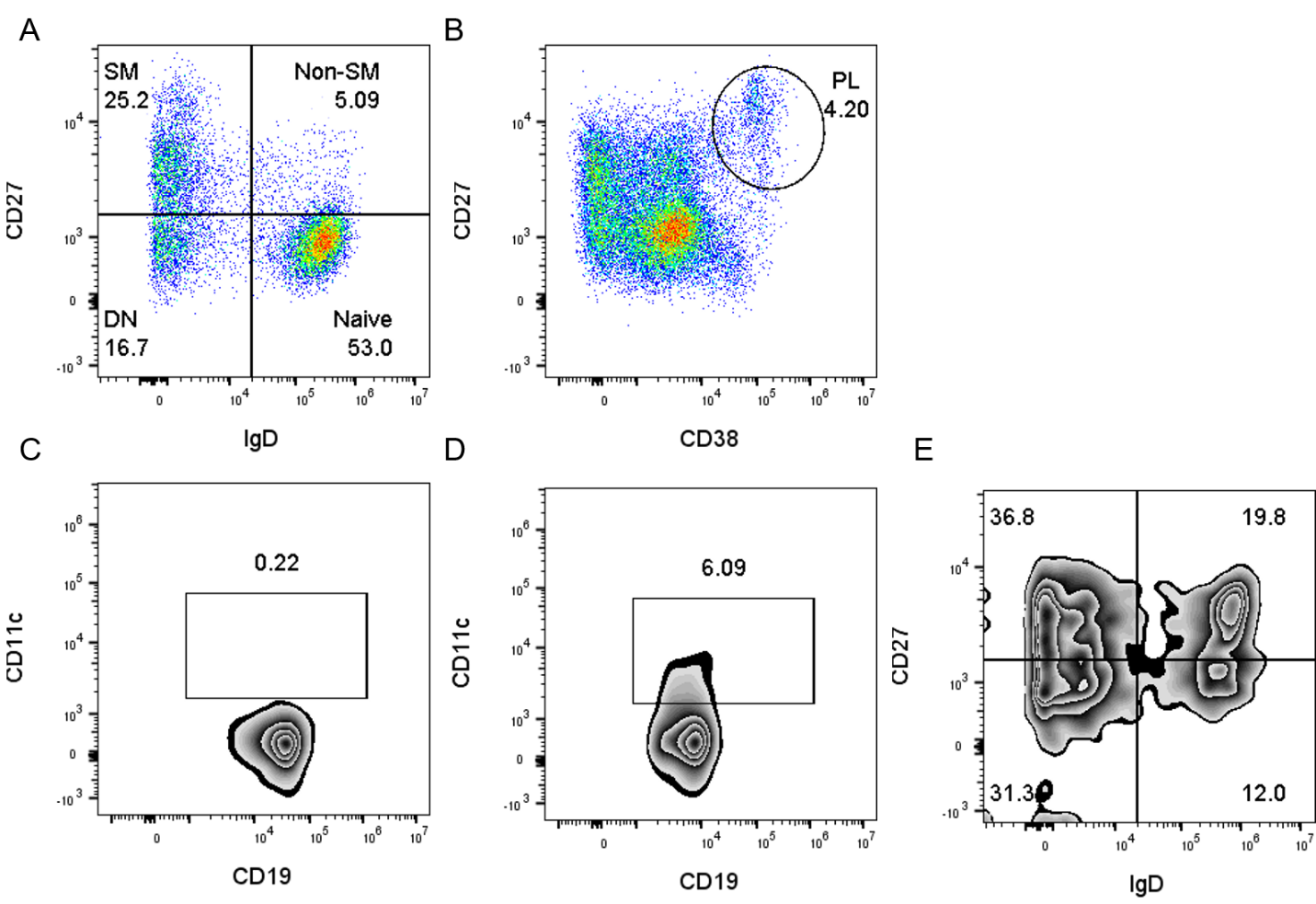
**

**Supplementary Figure 2: B cell phenotypes in patients with RA.** (A-B) Representative dot plots of B cell phenotypes on gated CD19+ B cells in patients with RA. (C-D) Representative flow analysis showing CD11c expression on gated CD19^+^IgD^-^CD27^-^ B cells. Plot (C) was the isotype control of CD11c antibody stained cells, and plot (D) was the CD11c antibody-stained cells.

**
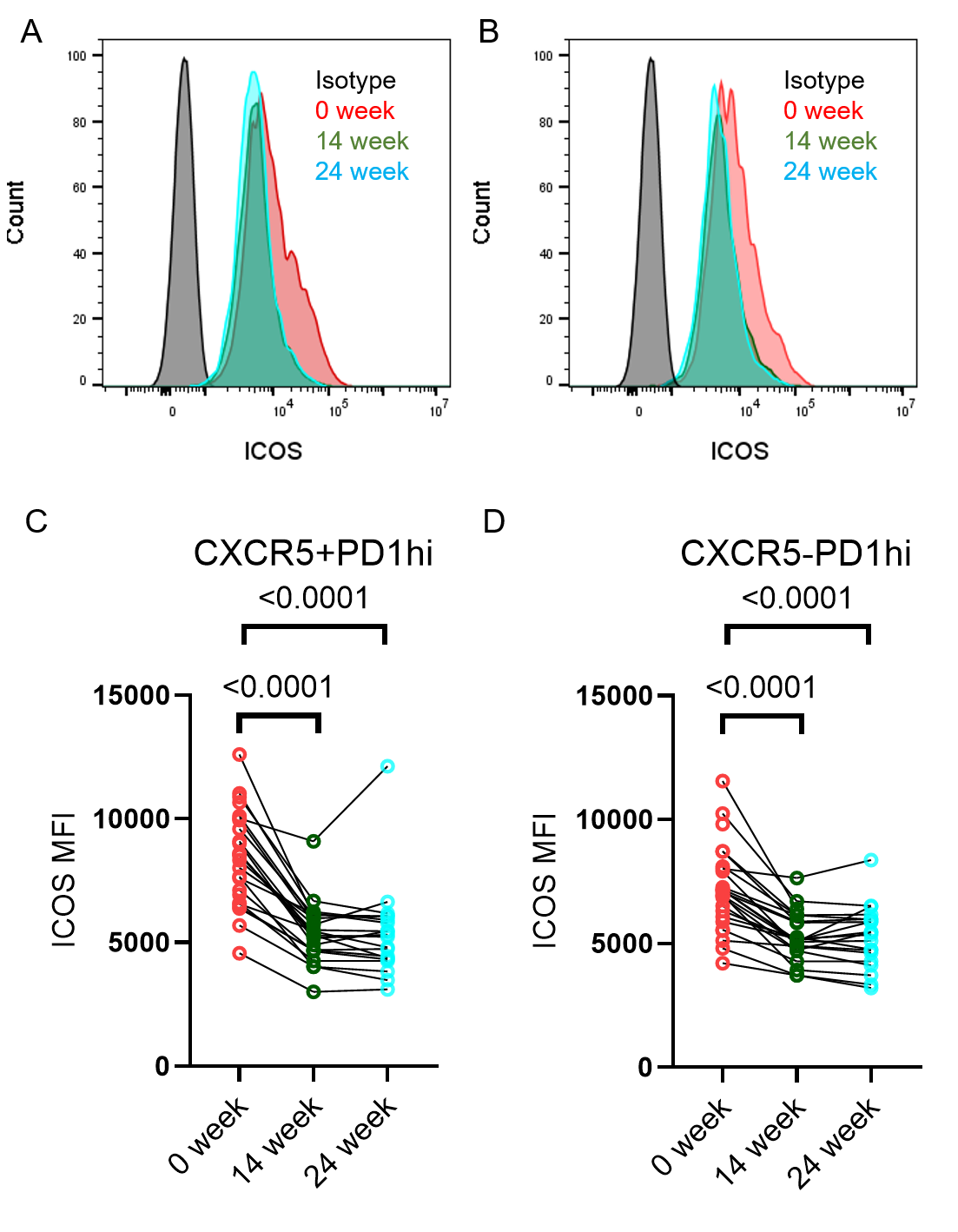
**

**Supplementary Figure 3: Changes of ICOS expression on Tfh and Tph cells with treatment of Abatacept in RA patients.** Representative histograms of ICOS expression on CXCR5+PD1hi Tfh cells (A) and CXCR5-PD1hi Tph cells (B) in RA patients. Changes of ICOS expression on CXCR5+PD1hi Tfh cells (C) and CXCR5-PD1hi Tph cells (D) in RA patients before and after abatacept treatment.

**
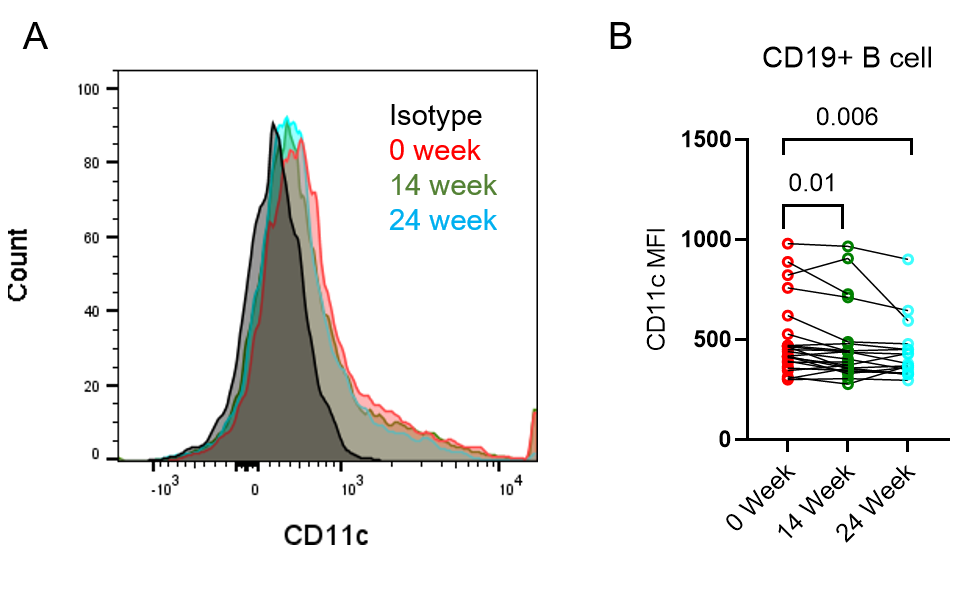
**

**Supplementary Figure 4: CD11c expression on CD19+ B cells with treatment of Abatacept in patients with RA.** Representative histograms of CD11c expression on CD19+ B cells (A). Changes of CD11c expression on CD19+ B cells in RA patients before and after abatacept treatment.
